# Sepsis survivors readmitted within 30 days: outcomes of a single-center retrospective study

**DOI:** 10.62675/2965-2774.20240116-en

**Published:** 2024-11-26

**Authors:** Abdelrahman Nanah, Fatima Abdeljaleel, Marcos Vinícius Fernandes Garcia, Kelly Pannikodu, Mohannad Seif, Amy Flowers-Surovi, Naveen Gopal, Divyajot Sadana

**Affiliations:** 1 Fairview Hospital Cleveland Clinic Foundation Department of Internal Medicine Cleveland Ohio United States Department of Internal Medicine, Cleveland Clinic Foundation, Fairview Hospital -Cleveland, Ohio, United States.; 2 Fairview Hospital Cleveland Clinic Foundation Department of Quality and Safety Cleveland Ohio United States Department of Quality and Safety, Cleveland Clinic Foundation, Fairview Hospital - Cleveland, Ohio, United States.

**Keywords:** Sepsis, Patient readmission, Quality improvement, Mortality, Critical care

## Abstract

**Objective:**

To investigate a cohort of sepsis survivors readmitted within 30 days postdischarge, explore the one-year mortality rate based on different causes of readmission and identify factors associated with increased one-year mortality risk among all sepsis survivors readmitted within this timeframe.

**Methods:**

This was a single-center retrospective cohort study involving adult sepsis survivors who were readmitted within 30 days of discharge. Patients were categorized into 3 groups based on the cause of readmission: same-source infectious readmission, different-source infectious readmission, and noninfectious readmission. The outcome of interest was all-cause one-year mortality. Cox proportional hazard analysis was performed to compare factors associated with one-year mortality.

**Results:**

Of the 1,666 patients admitted with sepsis, 243 (14.5%) were readmitted within 30 days. Readmissions were due to same-source infections (40.7%), different-source infections (21.4%), or noninfectious causes (37.9%). All-cause one-year mortality was 46.9%, with no difference between the groups. Age (HR 1.02; 95%CI: 1.003 - 1.04; p = 0.01), Sequential Organ Failure Assessment score (HR 1.1; 95%CI: 1.02 - 1.18; p = 0.01), discharge to a care facility during index admission (HR 1.6; 95%CI: 1.04 - 2.40; p = 0.03), and malignancy (HR 2.3; 95%CI: 1.5 - 3.7; p < 0.001) were associated with one-year mortality.

**Conclusion:**

Thirty-day readmission in sepsis survivors was common and was associated with a 46.9% one-year mortality rate regardless of readmission cause. Quality improvement patient safety initiatives based on local institutional factors may allow for targeted interventions to improve sepsis survivor outcomes.

## INTRODUCTION

Sepsis is a major healthcare concern. It is estimated to affect more than 30 million patients worldwide and contributes to more than 5.3 million deaths annually.^(1)^ When compounded by septic shock, the mortality rate can exceed 50%.^(2)^ While survival after sepsis has improved significantly in recent years,^(3,4)^ those patients are still at increased risk of long-term mortality and have high rates of healthcare use, including a 30-day readmission rate exceeding 20%.^(5–8)^ Sepsis remains one of the most common causes of hospital readmissions in the U.S., second only to congestive heart failure,^(9)^ prompting the Centers for Medicare & Medicaid Services (CMS) to utilize 30-day readmission rates as a metric for assessing the quality of care and as a reference for the financial compensation to institutions.^(10)^

In the context of a local multidisciplinary hospital-wide quality improvement initiative for sepsis care, we hypothesize that there are differences in long-term outcomes between infectious and noninfectious patients. We aimed to investigate a cohort of sepsis survivors readmitted within 30 days post-discharge, explore the one-year mortality rate based on different causes of readmission, and identify factors associated with increased one-year mortality risk among all sepsis survivors readmitted within this timeframe. Through this analysis, we aimed to uncover local patterns and inform targeted interventions to improve patient outcomes.

## METHODS

This single-center, retrospective cohort study was conducted at a 500-bed tertiary care center in Northeast Ohio between December 2021 and January 2023. We included adult patients (≥ 18 years) who were hospitalized with sepsis, discharged, and subsequently readmitted within 30 days of discharge.

All the data were extracted via chart review of electronic medical records (EMRs). Patients were categorized into three primary groups: readmission stemming from the same type of infectious source as the index admission (same-source infectious readmission), readmission arising from a different infectious source than the index admission (different-source infectious readmission), and noninfectious cause of readmission. The distinctions were made on the basis of clinical documentation, laboratory findings, and pertinent imaging reports, as appropriate.

Sepsis was identified through screening by the International Classification of Disease, 10^th^ edition (ICD-10) codes for sepsis, severe sepsis, or septic shock, as this has been found to provide a conservative estimate of sepsis incidence.^(11,12)^ To ensure the validity of the diagnosis, screened cases were then reviewed by experienced clinicians through the EMR, who confirmed the presence of sepsis based on the presence of a suspected or documented infection alongside organ dysfunction, denoted by an acute change in the Sequential Organ Failure Assessment (SOFA) score of ≥ 2, which is consistent with international guidelines.^(13,14)^

We collected demographic, clinical, and laboratory data from the hospital course during the index admission, readmission, and follow-up after discharge through a review of the EMR. In addition, we also collected data on the time to readmission, length of stay and mortality during readmission. If a patient experienced recurrent readmission, only the first readmission was considered. This study's outcome of interest was all-cause mortality within one year following discharge from a 30-day hospital readmission.

This study was approved by the Cleveland Clinic Foundation Institutional Review Board (protocol 23-621). This study followed the Strengthening Reporting of Observational Studies in Epidemiology (STROBE) reporting checklist for observational studies (Table 1S - Supplementary Material).^(15)^

### Statistical analysis

Categorical and continuous data are presented as counts (percentages) and medians [interquartile ranges (IQRs)], respectively. The chi-square test or Fisher's exact test was used to compare categorical variables, whereas the Mann–Whitney U or Kruskal–Wallis test was used to compare continuous variables, as appropriate. Survival curve analysis was conducted using the Kaplan–Meier method, with group comparisons performed via the log-rank test. A Cox proportional hazards model was employed to compare survival adjusted for covariates with a p value of < 0.1 in the univariate analysis between survivors and non-survivors at 1 year after discharge from readmission. The time to mortality was defined from the time of discharge from readmission, and patients who died prior to or during readmission were excluded from the analysis. All reported p values are two-sided, with statistical significance set at a value of less than 0.05. To evaluate the performance of the Cox model, discrimination was measured by the area under the receiver operating characteristic (ROC) curve. The statistical analysis was performed using R Studio for Windows (version 4.3.2).^(16)^

## RESULTS

**Figure 1:**
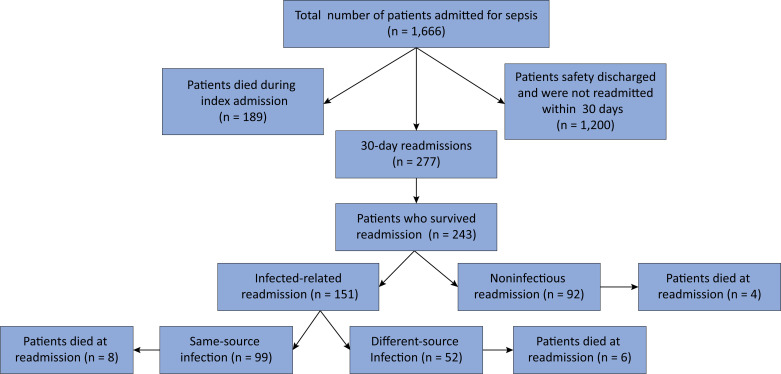
Patient inclusion flowchart.

Among the 1,666 patients admitted with sepsis, 243 (14.5%) were readmitted within 30 days of discharge (Figure 1). Readmissions stemmed from infectious causes in 151 (62.1%) patients and from noninfectious causes in 92 (37.9%) patients. Among the patients readmitted due to infection, 99 (65.6%) had a same-source infection, and 52 (34.4%) had different-source infections. The demographic factors, hospital course, and disposition of the readmitted patients are presented in table 1.

**Table 1:** Outcomes and predictors of one-year mortality in sepsis survivors following 30-day readmission

Variables		All patients (n = 243)	Infectious readmission: same source (n = 99)	Infectious readmission: different source (n = 52)	Noninfectious readmission (n = 92)	p value
Demographics						
	Age (years)	70 (60.5 - 78)	70 (60.5 - 77.5)	74.5 (60.8 - 80)	68 (60.8 - 77.3)	0.55
	Male gender	126 (51.9)	50 (55.6)	31 (59.6)	45 (48.9)	0.43
	Nonwhite race	60 (24.7)	23 (23.2)	19 (36.5)	18 (19.6)	0.06
Comorbidities						
	Heart failure	64 (26.3)	25 (25.3)	15 (28.8)	24 (26.1)	0.89
	Chronic obstructive pulmonary disease	80 (32.9)	35 (35.4)	16 (30.8)	29 (31.5)	0.79
	Hypertension	173 (71.2)	66 (66.7)	41 (78.9)	66 (71.7)	0.28
	Chronic kidney disease	74 (30.5)	27 (27.3)	18 (34.6)	29 (31.5)	0.62
	Cirrhosis	17 (7)	3(3)	5 (9.6)	9 (9.8)	0.13
	Active malignancy	38 (15.6)	11 (11.1)	12 (23.1)	15 (16.3)	0.15
	Diabetes mellitus	112 (46)	46 (46.5)	24 (46.2)	42 (45.7)	0.99
	Obesity	86 (35.4)	33 (33.3)	12 (23.1)	41 (44.6)	0.02
	Dementia	27 (11.1)	16 (16.2)	3 (5.8)	8 (8.7)	0.1
Index admission course						
	SOFA score	5 (3 - 8)	5 (4 - 8)	6 (4 - 9)	5 (3 - 8)	0.39
	Positive blood cultures	81 (33.3)	32 (32.3)	20 (38.5)	29 (31.5)	0.67
	ICU admission	136 (56)	61 (61.6)	25 (48.1)	50 (54.3)	0.26
	Shock	79 (32.5)	38 (38.4)	18 (34.6)	23 (25)	0.13
	Mechanical ventilation	33 (13.9)	13 (13.1)	12 (23.1)	8 (8.7)	0.053
	Use of steroids	43 (17.7)	18 (18.2)	7 (13.5)	18 (19.6)	0.64
	Length of stay (days)	7 (5 - 10)	7 (5 - 10)	8 (5.75 - 12)	7 (5 - 10)	0.35
	Antibiotics prescription on discharge	149 (61.3)	62 (62.6)	34 (65.4)	53 (57.6)	0.61
	Discharge directly from ICU	12 (4.9)	6 (6.1)	2 (3.8)	4 (4.3)	0.79
	Discharge to a care facility	111 (45.7)	53 (53.5)	27 (51.9)	31 (33.69)	0.01
Post discharge course						
	Time to readmission (days)	11 (6 - 16)	11 (6 - 16.5)	9.5 (4.7 - 15.7)	11 (5 - 19.2)	0.69
	Length of stay (readmission) (days)	6 (3 - 7)	6 (3 - 8)	6 (3 - 9.5)	4 (2 - 7)	0.002

SOFA - Sequential Organ Failure Assessment; ICU - intensive care unit. Comparison of the demographic factors, hospital course, and disposition plan in patients readmitted with same-source infection, different-source infection, and noninfectious admission. The results are expressed as the median (interquartile range) or n (%).

**Figure 2:**
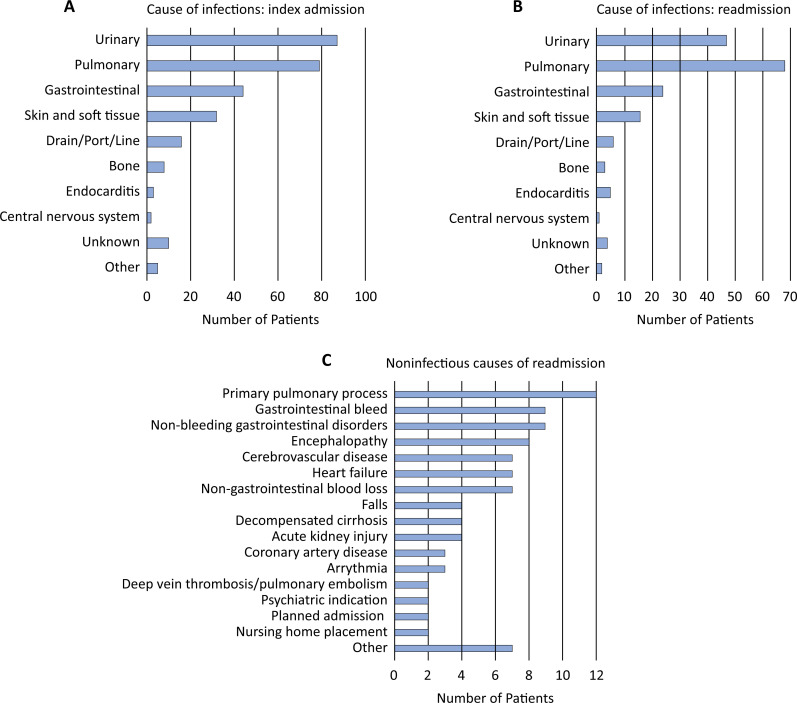
Column charts outlining the most common cause of infections at index admission (2A), readmission (2B), and the most common noninfectious causes of readmission (2C) (primary pulmonary processes include chronic obstructive pulmonary disease exacerbations, tracheostomy displacements, pleural effusions, and worsening obstructive hypoventilation syndrome, nonbleeding gastrointestinal disorders include gastroparesis, viral gastroenteritis, small bowel obstruction, fecal impaction, duodenitis, and increased ileostomy output).

The most common infectious causes on index admission were urinary tract infections, followed by pulmonary infections, gastrointestinal infections, and skin/soft tissue infections (Figure 2A). Upon readmission, the most frequent infectious sources were pulmonary, followed by the urinary tract, gastrointestinal tract, and skin/soft tissue (Figure 2B). *Clostridioides difficile* infection was noted in 4 (1.6%) patients. Noninfectious causes of readmission were variable (Figure 2C).

When demographic factors were compared among the three groups, most variables were not significantly different. However, obesity was more prevalent in patients readmitted for noninfectious causes. All 3 groups had a similar index hospital course, with comparable rates of SOFA scores, positive blood cultures, intensive care unit (ICU) admission, and length of stay. At discharge from index admission, all 3 groups received similar outpatient antibiotic prescriptions, with discharge to a care facility being more prevalent in patients readmitted with infectious concerns (Table 1).

The mortality rate at readmission was 7.4% for all patients, with no difference among readmissions from same-source infections (8%), different-source infections (7.7%), and noninfectious readmissions (6.5%; p = 0.91). Similarly, the time to readmission was similar across all groups [11 (6 - 16.5) *versus* 9.5 (4.8 - 15.8) *versus* 11 (5 - 19.3) days; p = 0.69]. Patients with noninfectious readmission were more likely to have a shorter length of stay at readmission [4 (2 - 7) days] than same-source [6 (3 - 8) days] and different-source infectious readmissions [6 (3 - 9.5) days; p = 0.002].

**Figure 3:**
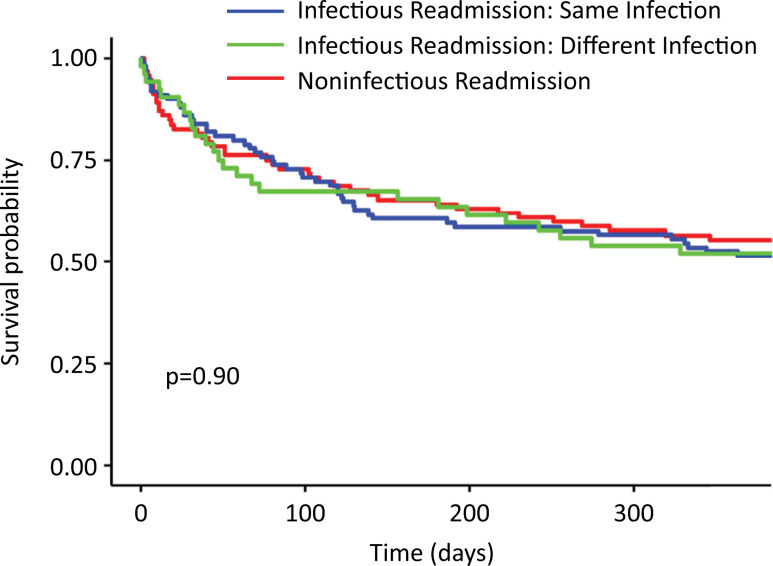
Kaplan–Meier survival plot for patients with same-source infectious readmission, different-source infectious readmission, and noninfectious readmission.

Within one year of discharge from readmission, 114 (46.9%) patients died, with no difference in mortality noted between readmissions from same-source infections (48.4%), different-source infections (48%), and noninfectious readmissions (44.6%; p = 0.84) (Figure 3).

When comparisons were made among the survivors and non-survivors, several differences were notable, including age [68 (55 - 76) *versus* 73.5 (63 - 81) years; p < 0.001], SOFA score [5 (3 - 7) *versus* 6 (4 - 9); p < 0.001], active malignancy (9.3% *versus* 22.8%; p = 0.006), dementia (7% *versus* 15.8%; p = 0.04), and discharge to a care facility on index admission (36.4% *versus* 71.9%; p = 0.003) (Table 2). On the basis of univariate analysis, a Cox proportional hazards model was generated with multivariable analysis adjusted for age, gender, SOFA score, infectious readmission, active malignancy, dementia, discharge to a care facility, time to readmission, and skin and soft tissue as the infectious source. This finding revealed an increased one-year mortality risk associated with age [hazard ratio (HR) 1.02 (95% confidence interval [95%CI]: 1.003 - 1.04; p = 0.01)], SOFA score (HR 1.1, 95%CI 1.02 - 1.18; p = 0.01), discharge to a care facility during the index admission (HR 1.6, 95%CI 1.04 - 2.40; p = 0.03), and active malignancy (HR 2.3, 95%CI 1.5 - 3.7; p < 0.001). An infectious cause of readmission was not associated with an increased risk of one-year mortality (Table 3). The Cox proportional model overall had an area under the curve of 0.7 (95%CI 0.6 - 0.7) (Figure 1S - Supplementary Material).

**Table 2:** Outcomes and predictors of one-year mortality in sepsis survivors following 30-day readmission

		Readmission survivors (n = 129)	Nonsurvivors (n = 114)	p value
Demographics				
	Age (years)	68 (55 - 76)	73.5 (63 - 81)	< 0.001
	Male gender	59 (45.7)	67 (58.8)	0.057
	Nonwhite race	29 (22.5)	31 (27.2)	0.48
Comorbidities				
	Heart failure	33 (25.6)	31 (27.2)	0.88
	Chronic obstructive pulmonary disease	41 (31.8)	39 (34.2)	0.79
	Hypertension	87 (67.4)	86 (75.4)	0.21
	Chronic kidney disease	37 (28.7)	37 (32.5)	0.61
	Cirrhosis	10 (7.8)	7 (6.1)	0.81
	Active malignancy	12 (9.3)	26 (22.8)	0.006
	Diabetes mellitus	57 (44.19)	55 (48.3)	0.61
	Obesity	51 (39.5)	35 (30.7)	0.19
	Dementia	9 (7)	18 (15.8)	0.04
Index admission course				
	SOFA score	5 (3 - 7)	6 (4 - 9)	< 0.001
	Positive blood cultures	37 (28.7)	43 (37.7)	0.21
	ICU admission	67 (51.9)	69 (60.5)	0.22
	Shock	35 (27.1)	44 (38.6)	0.07
	Mechanical ventilation	13 (10.1)	20 (17.5)	0.12
	Use of steroids	19 (14.7)	24 (21.1)	0.26
	Length of stay on index admission (days)	7 (5 - 10)	7 (5 - 12)	0.40
	Discharged directly from ICU	4 (3.1)	8 (7)	0.26
	Discharge to a care facility	47 (36.4)	82 (71.9)	0.003
Source of infection on index admission				
	Pulmonary	37 (28.7)	42 (36.8)	0.22
	Urinary	45 (34.9)	42 (36.8)	0.85
	Gastrointestinal	25 (19.4)	19 (16.7)	0.70
	Skin and soft tissue	22 (17.1)	10 (8.8)	0.08
	Bone	8 (6.2)	0 (0)	0.01
	Central nervous system	1 (0.8)	1 (0.9)	1
	Line, port, or drain	7 (5.4)	9 (7.9)	0.60
	Endocarditis	1 (0.8)	2 (1.8)	0.91
	Unknown	5 (3.9)	5 (4.4)	1
Postdischarge course				
	Length of stay on readmission (days)	5 (3 - 8)	5 (2.25 - 8.75)	0.89
	Time to readmission (days)	11 (6 - 21)	10 (5 - 15)	0.10
	Infectious cause of readmission	78 (60.5)	73 (64)	0.57

SOFA - Sequential Organ Failure Assessment; ICU - intensive care unit. Comparison of the demographic factors, hospital course, and disposition plan between sepsis survivors and nonsurvivors at one year. The results are expressed as the median (interquartile range) or n (%).

## DISCUSSION

In this retrospective cohort study of sepsis survivors, 30-day readmissions were common, affecting one in every six sepsis survivors. Readmissions were most commonly due to same-source infections as the index admissions, although more than 1/3 of readmissions occurred due to noninfectious causes.

The one-year mortality of readmitted patients was high at 46.9%, regardless of whether they had infectious or noninfectious readmissions, although the length of stay at readmission was shorter for noninfectious readmissions.

Sepsis diagnosis, management, and outcomes remain a focal point of intense research efforts.^(17–21)^ Our results are consistent with those of previous nationwide studies indicating that the readmission of sepsis survivors is common and morbid, most commonly occurring due to infectious etiologies.^(22–24)^ The incidence of 30-day readmission in sepsis survivors has varied from 17 to 28.7%,^(22,25,26)^ with risk factors include advanced age, male gender, nonwhite race, the presence of comorbidities, and discharge to a care facility.^(8)^

The most common cause of sepsis in our cohort was urinary tract infection. In contrast, epidemiological studies indicate that the pulmonary source of sepsis is the most common.^(27–29)^ We speculate that the prevalence of urosepsis in our cohort was due to local patient characteristics, including the advanced age of our cohort, a known risk factor for urinary tract infections.^(30)^ However, the definition of sepsis requires the presence of organ dysfunction, which may lead to overlap with other diagnoses, contributing to organ dysfunction in the context of bacterial colonization. Indeed, the diagnosis of sepsis poses many challenges related to the sensitivity of the diagnostic criteria.^(18,31)^

The infectious causes of readmission often stem from the same source as the index admission.^(32)^ The prognostic impact of infectious *versus* noninfectious causes of readmission in sepsis survivors remains poorly understood. Franco Palacios et al. reported that infectious causes of readmission were linked to a heightened risk of mortality.^(33)^ However, our cohort yielded different results. We observed a consistently high one-year mortality rate regardless of whether readmission stemmed from same-source infectious, different-source infectious, or noninfectious causes. We speculate that these differences may be due to the dynamic nature of sepsis, which is influenced by factors such as the host response and organ system involvement. Furthermore, our study cohort included all admitted patients who were readmitted within a 30-day timeframe, in contrast to the exclusive focus on ICU patients, with a median follow-up of 565 days reported by Franco Palacios et al. study.^(33)^

Predicting and preventing readmission in sepsis survivors presents significant challenges, regardless of the underlying cause of readmission. Readmission from the same source of infection can potentially be attributed to relapse, inadequate treatment of the initial infection, or the emergence of a new infection at the same site. Notably, research conducted by DeMerle et al. revealed that most same-site infectious readmissions were due to new infectious etiologies rather than relapses of untreated infections.^(32)^ This distinction holds considerable importance, as it impacts strategies for addressing source control and preventing readmissions. The relapse or recrudescence of infection may signal insufficient treatment as the primary concern, whereas the occurrence of new infections is often linked to a compromised host response and alterations in the gut microbiome, which are largely influenced by antibiotic usage.^(32,34,35)^ This finding is further supported by Sun et al., who identified a correlation between prolonged antibiotic use and increased risk of same-site infectious readmissions.^(36)^ Our dataset lacked sufficient granularity to determine whether same-site infectious readmissions were due to new or unresolved infections. Similarly, readmissions resulting from different-source infections, which accounted for one-fifth of all readmissions, may prove challenging to predict, except in cases such as *Clostridioides difficile* infections, which were infrequent in our cohort.

Readmissions from noninfectious causes present diverse etiologies, adding further complexity. Recent evidence indicates long-term cognitive and cardiovascular impairments in sepsis survivors, which contribute to worse mortality outcomes.^(5,37,38)^ Consequently, attempts to reduce readmission rates among sepsis survivors may encounter obstacles, with experts noting that readmission rates may not fully reflect the quality of patient care.^(39)^ Indeed, the prevention of readmission among sepsis survivors remains challenging, given the dynamic nature and frailty of this patient population. This challenge is underscored by a study involving over 20,000 sepsis survivors, which identified varying rates of readmission and mortality depending on factors such as hospital course, preexisting health conditions and functional status, discharge requirements, and healthcare accessibility.^(40)^

The factors associated with an increased risk of one-year mortality in our cohort were active malignancy, discharge to a care facility on index admission, increased SOFA scores, and increased age. These results echo previous findings indicating their association with elevated mortality risk among sepsis survivors.^(33,41–43)^ Notably, these factors represent nonmodifiable indicators of illness severity and functional deterioration.

Future directions to reduce readmissions and the associated patient burden include the provision of a sepsis transition of care team after a patient's discharge;^(44)^ appropriate utilization of telehealth, and coordinated discharge follow-up;^(45)^ and, in our cohort, timely goals-of-care discussions in patients with advanced comorbidities and active malignancies.

Strengths of our study include the close follow-up of our patient cohort and the comparison of distinct causes of readmission. This study is part of a quality improvement patient safety initiative to reduce readmission of sepsis survivors on the basis of local data.

Our study also has significant limitations, including the retrospective and single-center nature of the study and the inclusion of mostly White patients. In addition, we did not collect data on sepsis survivors who were not readmitted. Furthermore, the retrospective diagnosis of sepsis is challenging, with standardized scores such as the SOFA score being imperfect screening tools and more so markers of the severity of organ dysfunction.^(13,18)^ We attempted to mitigate these challenges by screening through ICD codes and confirming through individual EMR reviews. Finally, although we describe a comprehensive list of patient characteristics, standardized illness severity scores at admission (e.g., Charleston comorbidity index, acute physiology and chronic health evaluation score) are not routinely calculated at our institution, which may raise concerns about unmeasured patient characteristics affecting our outcomes.

## CONCLUSION

Thirty-day readmissions of sepsis survivors at our institution are common and are associated with a 46.9% one-year mortality rate regardless of the infectious or noninfectious cause of readmission. The factors associated with an increased risk of one-year mortality included active malignancy, increased SOFA score, discharge to a care facility on index admission, and advanced age. Additional research is warranted to ascertain whether quality improvement interventions tailored toward local institutional factors could mitigate the readmission rates of sepsis patients.

## Supplementary Material


