# Supplementary material for: Sepsis survivors readmitted within 30 days: outcomes of a single-center retrospective study
Source: Crit Care Sci. 2024 Nov 26;36:e20240116en. doi: 10.62675/2965-2774.20240116-en (PMC11812674; doi:10.62675/2965-2774.20240116-en)
Supplement: Supplementary file 1 [file 2965-2774-ccsci-36-e20240116en-Suppl01.pdf]

Critical Care Science
https://doi.org/10.62675/2965-2774.20240116-en

SUPPLEMENTARY MATERIAL

Sepsis survivors readmitted within 30 days: outcomes of a single-center retrospective study

Abdelrahman Nanah[1], Fatima Abdeljaleel[1], Marcos Vinícius Fernandes Garcia[1], Kelly Pannikodu[1], Mohannad Seif[1], Amy Flowers-Surovi[2], Naveen Gopal[1], Divyajot Sadana[1]

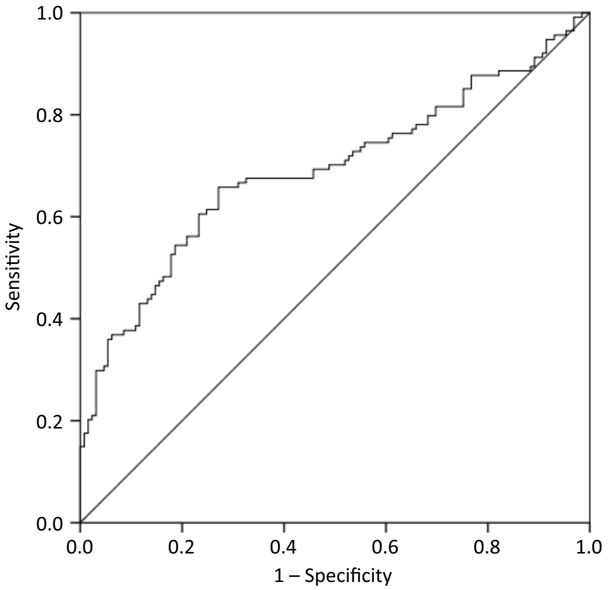

Area under curve	Standard error	Association level (alpha)	95% confidence interval
0.70	0.035	< 0.001	0.6 - 0.7

Figure 1S - Outcomes and predictors of one-year mortality in sepsis survivors following 30-day readmission.

This is an open access article under the CC BY license (https://creativecommons.org/licenses/by/4.0/).

Table 1S - Receiving operating characteristic analyzing the sensitivity and specificity of the Cox proportional hazard model

	Item nº	Recommendation	Page nº	Relevant text from manuscript
**Title and abstract**	1	(a) Indicate the study's design with a commonly used term in the title or the abstract	7	"Retrospective Cohort Study"
		(b) Provide in the abstract an informative and balanced summary of what was done and what was found	4	Structured abstract with methodology and findings
**Introduction**				
Background/rationale	2	Explain the scientific background and rationale for the investigation being reported	6	Quality improvement initiative for sepsis care
Objectives	3	State specific objectives, including any prespecified hypotheses	6	"We hypothesize there are differences in long-term outcomes between readmission causes"
**Methods**				
Study design	4	Present key elements of study design early in the paper	7	"Retrospective cohort study"
Setting	5	Describe the setting, locations, and relevant dates, including periods of recruitment, exposure, follow-up, and data collection	7	"Single Center" "Northeastern Ohio" "January 2021 to December 2022" "One-year follow up"
Participants	6	(a) Cohort study—Give the eligibility criteria, and the sources and methods of selection of participants. Describe methods of follow-up<br>Case-control study—Give the eligibility criteria, and the sources and methods of case ascertainment and control selection. Give the rationale for the choice of cases and controls<br>Cross-sectional study—Give the eligibility criteria, and the sources and methods of selection of participants	7	"Sepsis survivors readmitted within 30 days" "Sepsis defined as ...." "One-year follow up from date of index admission" "Electronic medical records"
		(b) Cohort study—For matched studies, give matching criteria and number of exposed and unexposed<br>Case-control study—For matched studies, give matching criteria and the number of controls per case		
Variables	7	Clearly define all outcomes, exposures, predictors, potential confounders, and effect modifiers. Give diagnostic criteria, if applicable	7	"Primary and secondary outcomes were..."
Data sources/measurement	8*	For each variable of interest, give sources of data and details of methods of assessment (measurement). Describe comparability of assessment methods if there is more than one group	8	Statistical Analysis paragraph
Bias	9	Describe any efforts to address potential sources of bias	8	"Multivariate analysis with AUC"
Study size	10	Explain how the study size was arrived at	7	All readmitted sepsis survivors were included
Quantitative variables	11	Explain how quantitative variables were handled in the analyses. If applicable, describe which groupings were chosen and why	8	Statistical Analysis Paragraph
Statistical methods	12	(a) Describe all statistical methods, including those used to control for confounding	8	Statistical Analysis Paragraph
		(b) Describe any methods used to examine subgroups and interactions	8	Statistical Analysis Paragraph
		(c) Explain how missing data were addressed	-	No missing data was identified
		(d) Cohort study—If applicable, explain how loss to follow-up was addressed<br>Case-control study—If applicable, explain how matching of cases and controls was addressed<br>Cross-sectional study—If applicable, describe analytical methods taking account of sampling strategy	8	Statistical Analysis Paragraph
		(e) Describe any sensitivity analyses	8	Statistical Analysis Paragraph
**Results**				
Participants	13*	(a) Report numbers of individuals at each stage of study—eg numbers potentially eligible, examined for eligibility, confirmed eligible, included in the study, completing follow-up, and analysed	9	Figure 1 Flowchart
		(b) Give reasons for non-participation at each stage	9	Figure 1 Flowchart
		(c) Consider use of a flow diagram	9	Figure 1 Flowchart

Continue...

...continuation

Descriptive data	14*	(a) Give characteristics of study participants (eg demographic, clinical, social) and information on exposures and potential confounders	9	Table 1
		(b) Indicate number of participants with missing data for each variable of interest	9	Table 1
		(c) Cohort study—Summarise follow-up time (eg, average and total amount)	9	Table 1
Outcome data	15*	Cohort study—Report numbers of outcome events or summary measures over time	9-10	Table 1-3
		Case-control study—Report numbers in each exposure category, or summary measures of exposure		
		Cross-sectional study—Report numbers of outcome events or summary measures		
Main results	16	(a) Give unadjusted estimates and, if applicable, confounder-adjusted estimates and their precision (eg, 95% confidence interval). Make clear which confounders were adjusted for and why they were included	9-10	Table 2-3
		(b) Report category boundaries when continuous variables were categorized	9-10	Mention of confidence intervals and p values
		(c) If relevant, consider translating estimates of relative risk into absolute risk for a meaningful time period	N/A	
Other analyses	17	Report other analyses done—eg analyses of subgroups and interactions, and sensitivity analyses	10	"Cox analysis AUC"
**Discussion**				
Key results	18	Summarize key results with reference to study objectives	11	Discussion introductory paragraph
Limitations	19	Discuss limitations of the study, taking into account sources of potential bias or imprecision. Discuss both direction and magnitude of any potential bias	13	Strengths and limitations paragraph
Interpretation	20	Give a cautious overall interpretation of results considering objectives, limitations, multiplicity of analyses, results from similar studies, and other relevant evidence	13	emphasis on local data, citation of previous publications
Generalizability	21	Discuss the generalisability (external validity) of the study results	13-14	Emphasis on local data
**Other information**				
Funding	22	Give the source of funding and the role of the funders for the present study and, if applicable, for the original study on which the present article is based	3	Funding statement in Cover page of manuscript

Strengthening the reporting of observational studies in epidemiology (STROBE) Statement—checklist of items that should be included in reports of observational studies.
