# Supplementary material for: Sepsis survivors readmitted within 30 days: outcomes of a single-center retrospective study
Source: Crit Care Sci. 2024 Nov 26;36:e20240116en. doi: 10.62675/2965-2774.20240116-en (PMC11812674; doi:10.62675/2965-2774.20240116-en)
Supplement: Translation

# Translation: Reinternação de pacientes sobreviventes à sepse em até 30 dias: desfechos de um estudo retrospectivo de centro único

**DOI:** 10.62675/2965-2774.20240006-pt

**Authors:** Abdelrahman Nanah, Fatima Abdeljaleel, Marcos Vinícius Fernandes Garcia, Kelly Pannikodu, Mohannad Seif, Amy Flowers-Surovi, Naveen Gopal, Divyajot Sadana

**Affiliations:** 1 Cleveland Ohio United States Department of Internal Medicine, Cleveland Clinic Foundation, Fairview Hospital -Cleveland, Ohio, United States.; 2 Cleveland Ohio United States Department of Quality and Safety, Cleveland Clinic Foundation, Fairview Hospital - Cleveland, Ohio, United States.

**Keywords:** Sepse, Reinternação do paciente, Melhoria de qualidade, Mortalidade, Cuidados críticos

## Abstract

**Objetivo:**

Investigar uma coorte de pacientes sobreviventes à sepse reinternados em até 30 dias após a alta hospitalar, explorar as taxas de mortalidade em 1 ano devido a diferentes causas de reinternação e identificar os fatores associados a um risco maior de mortalidade em 1 ano de todos os pacientes sobreviventes à sepse reinternados nesse período.

**Métodos:**

Trata-se de estudo de coorte retrospectivo de centro único de pacientes adultos com sepse reinternados em até 30 dias após a alta hospitalar. Os pacientes foram categorizados em três grupos conforme a causa da hospitalização: hospitalização infecciosa de mesma origem, hospitalização infecciosa de origem diferente e hospitalização não infecciosa. O desfecho de interesse foi a mortalidade por todas as causas em 1 ano. Realizou-se análise de risco proporcional de Cox para comparar os fatores associados à mortalidade em 1 ano.

**Resultados:**

Dos 1.666 pacientes internados com sepse, 243 (14,5%) foram reinternados em 30 dias. A reinternação ocorreu devido à mesma origem de infecção (40,7%), a uma infecção de origem diferente (21,4%) ou a causas não infecciosas (37,9%). A mortalidade por todas as causas em 1 ano foi de 46,9%, sem diferença entre os dois grupos. A idade (RR 1,02; IC95% 1,003 - 1,04; p = 0,01), a *Sequential Organ Failure Assessment* (RR 1,1; IC95% 1,02 - 1,18; p = 0,01), a alta para instituição de saúde na internação inicial (RR 1,6; IC95% 1,04 - 2,40; p = 0,03) e a malignidade (RR 2,3; IC95% 1,5 - 3,7; p < 0,001) foram associadas à mortalidade em 1 ano.

**Conclusão:**

A reinternação em 30 dias de sobreviventes à sepse é comum e está associada à taxa de mortalidade em 1 ano de 46,9%, independentemente da causa da reinternação. As iniciativas de melhoria da qualidade da segurança do paciente a partir de fatores institucionais locais podem permitir intervenções direcionadas para melhorar os desfechos de pacientes sobreviventes à sepse.

## INTRODUÇÃO

A sepse é uma das principais ameaças à saúde. Estima-se que ela afete mais de 30 milhões de pacientes em todo o mundo e contribua para mais de 5,3 milhões de mortes por ano.^(1)^ Quando agravada por choque séptico, a taxa de mortalidade pode exceder 50%.^(2)^ Embora a sobrevida após a sepse tenha melhorado significativamente nos últimos anos,^(3,4)^ esses pacientes ainda correm risco maior de mortalidade em longo prazo e têm altas taxas de uso de assistência médica, incluindo uma taxa de reinternação em 30 dias superior a 20%.^(5–8)^ A sepse continua sendo uma das causas mais comuns de reinternação hospitalar nos Estados Unidos, ficando em segundo lugar e perdendo apenas para a insuficiência cardíaca congestiva,^(9)^ levando os *Centers for Medicare & Medicaid Services* (CMS) a utilizarem as taxas de reinternação em 30 dias como métrica para a avaliação da qualidade dos cuidados e como referência para a compensação financeira das instituições.^(10)^

No contexto de uma iniciativa local multidisciplinar de melhoria da qualidade do tratamento da sepse em todo o hospital, levantamos a hipótese de que há diferenças nos desfechos em longo prazo entre pacientes com e sem infecção. Nosso objetivo foi investigar uma coorte de pacientes sobreviventes à sepse reinternados em até 30 dias após a alta hospitalar, explorar as taxas de mortalidade em 1 ano devido a diferentes causas de reinternação e identificar os fatores associados a um risco maior de mortalidade em 1 ano de todos os pacientes sobreviventes à sepse reinternados nesse período. Por meio desta análise, intentamos descobrir padrões locais e informar intervenções direcionadas para melhorar os desfechos dos pacientes.

## MÉTODOS

Trata-se de estudo de coorte retrospectivo de centro único conduzido em um centro de tratamento terciário com 500 leitos no nordeste de Ohio entre dezembro de 2021 e janeiro de 2023. Incluímos pacientes adultos (≥ 18 anos) hospitalizados com sepse, que receberam alta e, posteriormente, foram reinternados em até 30 dias após a alta.

Todos os dados foram extraídos por meio de revisão de prontuários médicos eletrônicos (PMEs). Os pacientes foram categorizados em três grupos principais: reinternação decorrente do mesmo tipo de fonte infecciosa que a internação inicial (reinternação infecciosa de mesma origem), reinternação decorrente de uma fonte infecciosa diferente da internação inicial (reinternação infecciosa de origem diferente) e causa não infecciosa de reinternação. As distinções foram feitas a partir da documentação clínica, dos achados laboratoriais e dos relatórios de imagens pertinentes, conforme os casos.

A sepse foi identificada por meio de triagem pelos códigos da Classificação Internacional de Doenças, décima edição (CID-10) para sepse, sepse grave ou choque séptico, pois se constatou que isso fornecia uma estimativa conservadora da incidência de sepse.^(11,12)^ Para garantir a validade do diagnóstico, os casos triados foram revisados por clínicos experientes por meio dos PMEs, os quais confirmaram a presença de sepse a partir da presença de uma infecção suspeita ou documentada com disfunção orgânica, denotada por uma alteração aguda na *Sequential Organ Failure Assessment* (SOFA) ≥ 2, consistente com as diretrizes internacionais.^(13,14)^

Coletamos dados demográficos, clínicos e laboratoriais da evolução hospitalar durante a internação inicial, reinternação e seguimento após a alta por meio de uma revisão dos PMEs. Além disso, também coletamos dados sobre o tempo até a reinternação, a duração da internação e a mortalidade durante a reinternação. No caso de reinternação recorrente de um paciente, considerou-se apenas a primeira reinternação. O desfecho de interesse deste estudo foi a mortalidade por todas as causas em até 1 ano após a alta de uma reinternação hospitalar de 30 dias.

Este estudo foi aprovado pelo Conselho de Revisão Institucional da *Cleveland Clinic Foundation* (protocolo 23-621). Este estudo seguiu a lista de verificação de relatórios para estudos observacionais *Strengthening Reporting of Observational Studies in Epidemiology* (STROBE) (Tabela 1S - Material Suplementar).^(15)^

### Análise estatística

Os dados categóricos e contínuos são apresentados como contagem (porcentagem) e mediana [intervalo interquartil (IIQ)], respectivamente. Utilizaram-se o teste do qui-quadrado ou o teste exato de Fisher para comparar variáveis categóricas, enquanto o teste U de Mann-Whitney ou o teste de Kruskal-Wallis foi utilizado para comparar variáveis contínuas, conforme o caso. O método Kaplan-Meier foi utilizado na análise da curva de sobrevida, com comparações de grupos realizadas por meio do teste *log-rank*. Um modelo de riscos proporcionais de Cox foi empregado para comparar a sobrevida ajustada de covariáveis com valor de p < 0,1 na análise univariada de sobreviventes e não sobreviventes em 1 ano após a alta da reinternação. O tempo até a mortalidade foi definido a partir do momento da alta da reinternação, sendo excluídos da análise os pacientes que morreram antes ou durante a reinternação. Todos os valores de p informados eram bilaterais, com significância estatística definida em valor menor que 0,05. Para avaliar o desempenho do modelo de Cox, a discriminação foi medida pela área sob a curva da característica de operação do receptor. A análise estatística foi realizada usando o R Studio para Windows (versão 4.3.2).^(16)^

## RESULTADOS

Dos 1.666 pacientes internados com sepse, 243 (14,5%) foram reinternados em até 30 dias após a alta (Figura 1). As reinternações foram decorrentes de fontes infecciosas em 151 (62,1%) pacientes e de fontes não infecciosas em 92 (37,9%) pacientes. Dos pacientes reinternados devido a infecção, 99 (65,6%) apresentavam a mesma origem de infecção, e 52 (34,4%) apresentavam infecções de origem diferente. Os fatores demográficos, a evolução hospitalar e a disposição dos pacientes reinternados são apresentados na tabela 1.

**Figura 1:** Fluxograma de inclusão de pacientes.

**Tabela 1:** Desfechos e preditores de mortalidade em 1 ano em sobreviventes à sepse após reinternação em 30 dias

Variáveis		Todos os pacientes (n = 243)	Reinternação infecciosa: mesma origem (n = 99)	Reinternação infecciosa: origem diferente (n = 52)	Reinternação não infecciosa (n = 92)	Valor de p
Dados demográficos						
	Idade (anos)	70 (60,5 - 78)	70 (60,5 - 77,5)	74,5 (60,8 - 80)	68 (60,8 - 77,3)	0,55
	Sexo masculino	126 (51,9)	50 (55,6)	31 (59,6)	45 (48,9)	0,43
	Cor não branca	60 (24,7)	23 (23,2)	19 (36,5)	18 (19,6)	0,06
Comorbidades						
	Insuficiência cardíaca	64 (26,3)	25 (25,3)	15 (28,8)	24 (26,1)	0,89
	Doença pulmonar obstrutiva crônica	80 (32,9)	35 (35,4)	16 (30,8)	29 (31,5)	0,79
	Hipertensão	173 (71,2)	66 (66,7)	41 (78,9)	66 (71,7)	0,28
	Doença renal crônica	74 (30,5)	27 (27,3)	18 (34,6)	29 (31,5)	0,62
	Cirrose	17 (7)	3(3)	5 (9,6)	9 (9,8)	0,13
	Malignidade ativa	38 (15,6)	11 (11,1)	12 (23,1)	15 (16,3)	0,15
	*Diabetes mellitus*	112 (46)	46 (46,5)	24 (46,2)	42 (45,7)	0,99
	Obesidade	86 (35,4)	33 (33,3)	12 (23,1)	41 (44,6)	0,02
	Demência	27 (11,1)	16 (16,2)	3 (5,8)	8 (8,7)	0,1
Evolução da internação inicial						
	SOFA	5 (3 - 8)	5 (4 - 8)	6 (4 - 9)	5 (3 - 8)	0,39
	Hemoculturas positivas	81 (33,3)	32 (32,3)	20 (38,5)	29 (31,5)	0,67
	Internação na UTI	136 (56)	61 (61,6)	25 (48,1)	50 (54,3)	0,26
	Choque	79 (32,5)	38 (38,4)	18 (34,6)	23 (25)	0,13
	Ventilação mecânica	33 (13,9)	13 (13,1)	12 (23,1)	8 (8,7)	0,053
	Uso de esteroides	43 (17,7)	18 (18,2)	7 (13,5)	18 (19,6)	0,64
	Duração da internação (dias)	7 (5 - 10)	7 (5 - 10)	8 (5,75 - 12)	7 (5 - 10)	0,35
	Prescrição de antibióticos na alta	149 (61,3)	62 (62,6)	34 (65,4)	53 (57,6)	0,61
	Alta diretamente da UTI	12 (4,9)	6 (6,1)	2 (3,8)	4 (4,3)	0,79
	Alta para uma instituição de saúde	111 (45,7)	53 (53,5)	27 (51,9)	31 (33,69)	0,01
Evolução após a alta						
	Tempo até a reinternação (dias)	11 (6 - 16)	11 (6 - 16,5)	9,5 (4,7 - 15,7)	11 (5 - 19,2)	0,69
	Duração da internação (reinternação) (dias)	6 (3 - 7)	6 (3 - 8)	6 (3 - 9,5)	4 (2 - 7)	0,002

SOFA - *Sequential Organ Failure Assessment*; UTI - unidade de terapia intensiva. Comparação dos fatores demográficos, evolução hospitalar e plano de disposição de pacientes reinternados com infecção de mesma origem, infecção de origem diferente e internação não infecciosa. Resultados expressos como mediana (intervalo interquartil) ou n (%).

As fontes infecciosas mais comuns na internação inicial foram infecções do trato urinário, seguidas de infecções pulmonares, gastrintestinais e de pele/tecidos moles (Figura 2A). Na reinternação, as fontes infecciosas mais frequentes foram pulmonares, seguidas pelo trato urinário, trato gastrintestinal e pele/tecido mole (Figura 2B). A infecção por *Clostridioides difficile* foi observada em quatro (1,6%) pacientes. As causas não infecciosas de reinternação foram variáveis (Figura 2C).

**Figura 2:** Gráficos de colunas descrevendo as causas mais comuns de infecção na internação inicial (2A), na reinternação (2B) e as causas não infecciosas mais comuns de reinternação (2C). Os processos pulmonares primários incluem exacerbações de doença pulmonar obstrutiva crônica. Os distúrbios gastrintestinais não hemorrágicos incluem gastroparesia, gastroenterite viral, obstrução do intestino delgado, impactação fecal, duodenite e aumento do débito da ileostomia.

Quando se compararam os fatores demográficos dos três grupos, a maioria das variáveis não apresentou diferença significativa. Entretanto, a obesidade foi mais prevalente em pacientes reinternados por causas não infecciosas. Todos os três grupos tiveram um percurso hospitalar semelhante, com taxas comparáveis de SOFA, hemoculturas positivas, internação na unidade de terapia intensiva (UTI) e duração da internação. Na alta da internação inicial, os três grupos receberam prescrições de antibióticos ambulatoriais semelhantes, sendo que a alta para uma unidade de saúde foi mais prevalente em pacientes reinternados com problemas infecciosos (Tabela 1).

A taxa de mortalidade na reinternação foi de 7,4% para todos os pacientes, sem diferença entre reinternações por infecções de mesma origem (8%), infecções de origem diferente (7,7%) e reinternações não infecciosas (6,5%; p = 0,91). Da mesma forma, o tempo de reinternação foi semelhante em todos os grupos [11 (6 - 16,5) *versus* 9,5 (4,8 - 15,8) *versus* 11 (5 - 19,3) dias; p = 0,69]. Os pacientes com reinternação não infecciosa eram mais propensos a ter uma duração de internação mais curta na reinternação [4 (2 - 7) dias] do que os pacientes com reinternação de mesma origem [6 (3 - 8) dias] e reinternação infecciosa de causa diferente [6 (3 - 9,5) dias; p = 0,002].

Em até 1 ano após a alta da reinternação, 114 (46,9%) pacientes morreram, sem diferença na mortalidade observada entre reinternações por infecções de mesma origem (48,4%), infecções de origem diferente (48%) e reinternações não infecciosas (44,6%; p = 0,84) (Figura 3).

**Figura 3:** Gráfico Kaplan-Meier de sobrevida de pacientes com reinternação infecciosa de mesma origem, reinternação infecciosa de origem diferente e reinternação não infecciosa.

Nas comparações feitas entre sobreviventes e não sobreviventes, várias diferenças foram notáveis, incluindo idade [68 (55 - 76) *versus* 73,5 (63 - 81) anos; p < 0,001], SOFA [5 (3 - 7) *versus* 6 (4 - 9); p < 0,001], malignidade ativa (9,3% *versus* 22,8%; p = 0,006), demência (7% *versus* 15,8%; p = 0,04) e alta para uma instituição de saúde na internação inicial (36,4% *versus* 71,9%; p = 0,003) (Tabela 2). A análise univariada permitiu gerar um modelo de riscos proporcionais de Cox com análise multivariável ajustada para idade, sexo, SOFA, reinternação infecciosa, malignidade ativa, demência, alta para um centro de saúde, tempo para reinternação e pele e tecidos moles como fonte infecciosa. Esse achado revelou aumento no risco de mortalidade em 1 ano associado à idade [razão de risco (RR) de 1,02 (intervalo de confiança de 95% [IC95%] 1,003 - 1,04; p = 0,01)], SOFA (RR 1,1, IC95% 1,02 - 1,18; p = 0,01), alta para uma instituição de saúde durante a internação inicial (RR 1,6, IC95% 1,04 - 2,40; p = 0,03) e malignidade ativa (RR 2,3, IC95% 1,5 - 3,7; p < 0,001). Nenhuma causa infecciosa de reinternação foi associada a um risco maior de mortalidade em 1 ano (Tabela 3). O modelo proporcional de Cox em geral teve área sob a curva de 0,7 (IC95% 0,6 - 0,7) (Figura 1S - Material Suplementar).

**Tabela 2:** Desfechos e preditores de mortalidade em 1 ano em sobreviventes à sepse após reinternação em 30 dias

		Sobreviventes à reinternação (n = 129)	Não sobreviventes (n = 114)	Valor de p
	Dados demográficos			
	Idade (anos)	68 (55 - 76)	73,5 (63 - 81)	< 0,001
	Sexo masculino	59 (45,7)	67 (58,8)	0,057
	Cor não branca	29 (22,5)	31 (27,2)	0,48
Comorbidades				
	Insuficiência cardíaca	33 (25,6)	31 (27,2)	0,88
	Doença pulmonar obstrutiva crônica	41 (31,8)	39 (34,2)	0,79
	Hipertensão	87 (67,4)	86 (75,4)	0,21
	Doença renal crônica	37 (28,7)	37 (32,5)	0,61
	Cirrose	10 (7,8)	7 (6,1)	0,81
	Malignidade ativa	12 (9,3)	26 (22,8)	0,006
	*Diabetes mellitus*	57 (44,19)	55 (48,3)	0,61
	Obesidade	51 (39,5)	35 (30,7)	0,19
	Demência	9 (7)	18 (15,8)	0,04
Evolução da internação inicial				
	SOFA	5 (3 - 7)	6 (4 - 9)	< 0,001
	Hemoculturas positivas	37 (28,7)	43 (37,7)	0,21
	Internação na UTI	67 (51,9)	69 (60,5)	0,22
	Choque	35 (27,1)	44 (38,6)	0,07
	Ventilação mecânica	13 (10,1)	20 (17,5)	0,12
	Uso de esteroides	19 (14,7)	24 (21,1)	0,26
	Duração da internação inicial (dias)	7 (5 - 10)	7 (5 - 12)	0,40
	Alta diretamente da UTI	4 (3,1)	8 (7)	0,26
	Alta para uma instituição de saúde	47 (36,4)	82 (71,9)	0,003
Origem da infecção na internação inicial				
	Pulmonar	37 (28,7)	42 (36,8)	0,22
	Urinária	45 (34,9)	42 (36,8)	0,85
	Gastrintestinal	25 (19,4)	19 (16,7)	0,70
	Pele e tecidos moles	22 (17,1)	10 (8,8)	0,08
	Óssea	8 (6,2)	0 (0)	0,01
	Sistema nervoso central	1 (0,8)	1 (0,9)	1
	Linha, porta ou dreno	7 (5,4)	9 (7,9)	0,60
	Endocardite	1 (0,8)	2 (1,8)	0,91
	Desconhecida	5 (3,9)	5 (4,4)	1
Evolução após a alta				
	Duração da internação na reinternação (dias)	5 (3 - 8)	5 (2,25 - 8,75)	0,89
	Tempo até a reinternação (dias)	11 (6 - 21)	10 (5 - 15)	0,10
	Causa infecciosa da reinternação	78 (60,5)	73 (64)	0,57

SOFA - *Sequential Organ Failure Assessment*; UTI - unidade de terapia intensiva. Comparação dos fatores demográficos, evolução hospitalar e plano de disposição de pacientes sobreviventes e não sobreviventes à sepse em 1 ano. Resultados expressos como mediana (intervalo interquartil) ou n (%).

## DISCUSSÃO

Neste estudo de coorte retrospectivo de sobreviventes à sepse, foram frequentes as reinternações em 30 dias, afetando um em cada seis sobreviventes à sepse. As reinternações foram mais comumente causadas por infecções de mesma origem que as internações iniciais, embora mais de um terço das reinternações tenha ocorrido devido a causas não infecciosas.

A mortalidade em 1 ano dos pacientes reinternados foi alta, de 46,9%, independentemente de terem tido reinternações infecciosas ou não infecciosas, embora a duração da internação na reinternação tenha sido menor nas reinternações não infecciosas.

O diagnóstico, o tratamento e os desfechos da sepse continuam um ponto focal de intensos esforços de pesquisa.^(17–21)^ Nossos resultados são consistentes com os de estudos nacionais anteriores que indicam que a reinternação de sobreviventes à sepse é comum e mórbida, ocorrendo mais comumente devido a etiologias infecciosas.^(22–24)^ A incidência de reinternação em 30 dias em sobreviventes à sepse variou de 17 a 28,7%,^(22,25,26)^ sendo que os fatores de risco incluem idade avançada, sexo masculino, cor da pele não branca, presença de comorbidades e alta para uma unidade de saúde.^(8)^

A causa mais comum de sepse em nossa coorte foi a infecção do trato urinário. Por outro lado, estudos epidemiológicos indicam que a causa pulmonar de sepse é a mais comum.^(27–29)^ Especulamos que a prevalência de sepse urinária em nossa coorte se deveu às características locais dos pacientes, incluindo a idade avançada de nossa coorte, que é um fator de risco conhecido de infecções do trato urinário.^(30)^ No entanto, a definição de sepse requer a presença de disfunção orgânica, o que pode levar à sobreposição com outros diagnósticos, contribuindo para a disfunção orgânica no contexto da colonização bacteriana. De fato, o diagnóstico de sepse apresenta muitos desafios relacionados à sensibilidade dos critérios diagnósticos.^(18,31)^

As fontes infecciosas de reinternação geralmente têm a mesma origem que a internação inicial.^(32)^ O impacto prognóstico das fontes infecciosas e não infecciosas de reinternação em sobreviventes à sepse permanece pouco conhecido. Franco Palacios et al. relataram que as fontes infecciosas de reinternação estavam ligadas a um maior risco de mortalidade.^(33)^ Entretanto, nossa coorte apresentou resultados diferentes. Observamos uma taxa de mortalidade consistentemente alta em 1 ano, independentemente de a reinternação ser decorrente de fontes infecciosas de mesma origem, infecciosas de origem diferente ou não infecciosas. Especulamos que essas diferenças podem ser devidas à natureza dinâmica da sepse, que é influenciada por fatores como a resposta do hospedeiro e o envolvimento do sistema de órgãos. Além disso, nossa coorte de estudo incluiu todos os pacientes internados que foram reinternados em um período de 30 dias, diferentemente do foco exclusivo em pacientes de UTI, com seguimento mediano de 565 dias relatado pelo estudo de Franco Palacios et al.^(33)^

A previsão e a prevenção da reinternação em sobreviventes à sepse apresentam desafios significativos, independentemente da causa subjacente da reinternação. A reinternação pela mesma fonte de infecção pode ser atribuída a uma recaída, a um tratamento inadequado da infecção inicial ou ao surgimento de uma nova infecção no mesmo local. Notavelmente, a pesquisa conduzida por DeMerle et al. revelou que a maioria das reinternações infecciosas no mesmo local foi causada por novas etiologias infecciosas, não por recaídas de infecções não tratadas.^(32)^ Essa distinção tem importância considerável, pois afeta as estratégias para abordar o controle da causa e evitar readmissões. A reincidência ou recrudescência da infecção pode sinalizar tratamento insuficiente como a principal questão, ao passo que a ocorrência de novas infecções geralmente está ligada a uma resposta comprometida do hospedeiro e a alterações no microbioma intestinal, que são amplamente influenciadas pelo uso de antibióticos.^(32,34,35)^ Essa constatação é apoiada ainda por Sun et al. que identificaram uma correlação entre o uso prolongado de antibióticos e o aumento do risco de reinternações infecciosas no mesmo local.^(36)^ Nosso conjunto de dados não tinha granularidade suficiente para determinar se as reinternações infecciosas no mesmo local eram devidas a infecções novas ou não resolvidas. Da mesma forma, as reinternações resultantes de infecções de origem diferente, que representaram um quinto de todas as readmissões, podem ser difíceis de prever, exceto em casos como infecções por *C. difficile*, que não eram frequentes em nossa coorte.

As reinternações por causas não infecciosas apresentam diversas etiologias, o que aumenta a complexidade. Evidências recentes indicam deficiências cognitivas e cardiovasculares de longo prazo em sobreviventes à sepse, o que contribui para desfechos de mortalidade menos favoráveis.^(5,37,38)^ Consequentemente, as tentativas de reduzir as taxas de reinternação de sobreviventes à sepse podem encontrar obstáculos; especialistas observam que as taxas de reinternação podem não refletir totalmente a qualidade da assistência ao paciente.^(39)^ De fato, a prevenção da reinternação de sobreviventes à sepse continua um desafio, dada a natureza dinâmica e a fragilidade dessa população de pacientes. Esse desafio é ressaltado por um estudo que envolveu mais de 20 mil sobreviventes à sepse e que identificou taxas variáveis de reinternação e mortalidade, dependendo de fatores como curso hospitalar, condições de saúde preexistentes e estado funcional, requisitos de alta e acessibilidade ao sistema de saúde.^(40)^

Os fatores associados a um risco maior de mortalidade em 1 ano em nossa coorte foram malignidade ativa, alta para uma instituição de saúde na internação inicial, aumento dos escores SOFA e aumento da idade. Esses resultados repercutem achados anteriores que indicam sua associação com o risco elevado de mortalidade de sobreviventes à sepse.^(33,41–43)^ Notavelmente, esses fatores representam indicadores não modificáveis de gravidade da doença e deterioração funcional.

As perspectivas futuras para reduzir as reinternações e o ônus associado ao paciente incluem o fornecimento de uma equipe de transição de cuidados com a sepse após a alta do paciente;^(44)^ a utilização adequada da telessaúde e seguimento coordenado da alta;^(45)^ e, em nossa coorte, discussões oportunas das metas de cuidados em pacientes com comorbidades avançadas e doenças malignas ativas.

Os pontos fortes de nosso estudo incluem o seguimento rigoroso de nossa coorte de pacientes e a comparação de causas distintas de reinternação. Este estudo faz parte de uma iniciativa de melhoria da qualidade e da segurança do paciente para reduzir a reinternação de sobreviventes à sepse a partir de dados locais.

Nosso estudo também tem limitações significativas, incluindo a natureza retrospectiva e de centro único do estudo e a inclusão principalmente de pacientes brancos. Além disso, não coletamos dados sobre sobreviventes à sepse que não foram reinternados. O diagnóstico retrospectivo da sepse também é desafiador, sendo que os escores padronizados, como o escore SOFA, são ferramentas de triagem imperfeitas e, mais ainda, marcadores da gravidade da disfunção orgânica.^(13,18)^ Tentamos mitigar esses desafios fazendo a triagem por meio de códigos da CID e confirmando por revisões individuais de PMEs. Por fim, apesar de descrevermos uma lista abrangente de características do paciente, os escores padronizados de gravidade da doença na internação (por exemplo: índice de comorbidade de Charleston, fisiologia aguda e escore de avaliação de saúde crônica) não são calculados rotineiramente em nossa instituição, o que pode levantar preocupações acerca de características não mensuradas do paciente que afetam nossos desfechos.

## CONCLUSÃO

As reinternações de sobreviventes à sepse em 30 dias em nossa instituição são comuns e estão associadas à taxa de mortalidade em 1 ano de 46,9%, independentemente de a causa da reinternação ser infecciosa ou não infecciosa. Os fatores associados ao aumento do risco de mortalidade em 1 ano incluíram malignidade ativa, aumento da SOFA, alta para uma instituição de saúde na internação inicial e idade avançada. Justifica-se a realização de mais pesquisas para verificar se as intervenções de melhoria da qualidade adaptadas aos fatores institucionais locais poderiam reduzir as taxas de reinternação de pacientes com sepse.
